# Is there a Role of Intravenous Immunoglobulin in Immunologic Recurrent Pregnancy Loss?

**DOI:** 10.1155/2020/6672865

**Published:** 2020-12-27

**Authors:** Xiuhua Yang, Tao Meng

**Affiliations:** Department of Obstetrics, The First Hospital of China Medical University, Shenyang 110001, China

## Abstract

Recurrent pregnancy loss (RPL) commonly refers to three or more miscarriages that occur before 20 weeks of pregnancy. The immunological cause of RPL could be either an auto- or alloimmune-related event or both. Because of the discovery of immunological abnormalities in RPL patients in clinical practice, several immunomodulatory therapies were introduced to maintain the immune balance at the maternal-fetal interface. Intravenous immunoglobulin (IVIg) is one of the immunomodulators. In recent years, several studies have analyzed the therapeutic effect of IVIg on RPL patients with antiphospholipid syndrome (APS) or unexplained RPL. However, their results are controversial. IVIg can be used in RPL patients with APS who have previously failed in other treatments. It is recommended that IVIg infusion could be considered used before conception in RPL patients who have cellular immune abnormalities such as increased natural killer (NK) cell counts, NK cell cytotoxicity, or increased T helper (Th)1/Th2 ratio, depending on the cut-off values of each hospital. The aim of this review was to summarize the mechanisms, efficacy, pharmacokinetics, and side effects associated with passive immunization using IVIg in immunologic RPL, according to the literature published in recent years. We hope that more obstetricians will be able to understand the timing and indication of IVIg properly in immunologic RPL patients and effectively enhance pregnancy outcomes for mothers and neonates.

## 1. Introduction

Recurrent pregnancy loss (RPL) commonly refers to three or more miscarriages that occur before 20 weeks of pregnancy [1]. The American Society for Reproductive Medicine also considers it to be two or more times [2]. The incidence of RPL in couples of childbearing age is 5% [3]. It can be divided into two types: primary RPL (previously no child) and secondary RPL (consecutive abortions after a live child). The etiology of RPL includes genetic, environmental, anatomical, endocrine, immune, and microbial infection factors [4–6]. However, nearly half of the RPL patients are called unexplained RPL due to unknown etiology. After a detailed analysis of these unexplained RPL patients, these women are often combined with immune abnormalities, i.e., abnormal immune tolerance at the maternal-fetal interface [7].

The immunological cause of RPL could be either an auto- or alloimmune-related event or both. Autoimmune abortion refers to the fact that there are autoimmune antibodies in the maternal body, such as anti-phospholipid antibodies (APAs), which kill decidual or trophoblast cells, thus affecting the development of the placenta and fetus [8, 9]. APAs occur in one fifth of patients with RPL [10]. Alloimmune miscarriages refer to the impairment of the maternal alloimmune response to paternally generated molecules on trophoblasts leading to unacceptability of the semiallogeneic fetus [11, 12]. Furthermore, many RPL patients have cellular immune abnormalities, including increased T helper (Th)1/Th2 cell ratios, higher natural killer (NK) cell counts or NK cytotoxicity, and aberrant regulatory T cell (Treg)/TH17 ratios [2–5, 7, 8, 13, 14].

Because of the discovery of immunological abnormalities in RPL patients in clinical practice, several immunomodulatory therapies were introduced to maintain the immune balance at the maternal-fetal interface. Intravenous immunoglobulin (IVIg) is one of the immunomodulators. It is extracted from the plasma of 3,000 or more donors and includes more than 95% unmodifed immunoglobulin G. IVIg is an important contributor to many autoimmune and inflammatory diseases [15–17]. The immunosuppressive properties of IVIg are as follows, including the downregulating of the functions of B cells [12], anti-idiotypic suppression of autoantibodies [18], reduction of Fc receptor-induced phagocytosis [19], adding the regulatory function of T cells [8], inhibiting the complement activation system [20], and controlling the expressions and functions of cytokines [21].

More than 20 years ago, IVIg was used for the first time in the prevention of RPL with a live birth rate of 80-82 percent [22, 23]. Likewise, Lee et al. reported that the live birth rate of RPL women with cellular immune abnormality who were treated with IVIg was 84.7%, which was similar to that of RPL women with normal cellular immunity who did not receive IVIg (89.7%) [24]. In recent years, several studies and randomized controlled trials have analyzed the therapeutic effect of IVIg on immunologic RPL patients. However, their results are controversial. Some studies have indeed shown that IVIg can significantly improve the pregnancy outcome of RPL patients [25–27], while other studies suggested that IVIg has no obvious therapeutic effect [28–30]. Therefore, the lack of sufficient evidence is the reason IVIg is not included in the clinical treatment guidelines for RPL [31]. This conclusion was mainly suggested by Daya et al. [32] and from the meta-analysis of six studies conducted by the German RSA/IVIG Group [33], Christiansen et al. [34], Stephenson et al. [35], Perino et al. [36], Coulam et al. [37], and Kipron et al. [38]. Different experimental results may be due to different RPL definitions. Some experiments have defined RPL more than two times, while others have defined it more than three times. RPL patients who have been treated with IVIg may have different types of immune abnormalities. In the meantime, clinicians may lack an in-depth understanding of the mechanism of IVIg in immunologic RPL. Understanding how IVIg might act to prevent immunologic abortions is important in selecting and assessing the efficiency of patients likely to benefit from this treatment. The aim of this review was to summarize the mechanisms, efficacy, pharmacokinetics, and side effects associated with passive immunization using IVIg in immunologic RPL. Two independent researchers searched the articles in Pubmed with the following medical subject headings (MeSH): “intravenous immunoglobulin,” “recurrent pregnancy loss,” “recurrent miscarriages,” “autoimmunity,” or “cellular immunity.” All articles were published in English from January 1994 to September 2020. We ruled out letters. We hope that more obstetricians will be able to understand the timing and indication of IVIg properly in immunologic RPL patients and effectively enhance pregnancy outcomes for mothers and neonates.

## 2. Mechanisms of IVIg in RPL

IVIg plays its immunomodulatory role through certain mechanisms of action that work together to exert a cooperative effect on RPL in the process of integration. The following possible theories may explain why passive IVIg immunotherapy can play an active role in the prevention of RPL (Table 1) [39–41].

## 3. IVIg in APS Patients with RPL

IVIg was used to prevent RPL associated with APS, and a better fetal outcome was reported, 70–100% [42–46]. In one report, 38 APS patients who had three or more early pregnancy abortions were included and received 300 mg/kg IVIg every three weeks from the determination of pregnancy to 16-17 weeks [46]. Out of the 38 patients, 34 women (89.4%) passed through the first trimester, and 31 females (81.4%) gave birth at full term [46]. In another study conducted by Triolo et al., 30 out of 40 patients received IVIg, 62.5% of the patients gave birth at 38-40 weeks of gestation, 12.5% gave birth at 29-35 weeks of gestation, and the remaining 25% had miscarriages [47]. In vivo IVIg has also been shown to be effective in preventing RPL and to reduce the rate of fetal abortion in murine RPL models associated with APS [28, 48].

In patients treated with IVIg, maternal and fetal outcomes were better than those who were treated with low-dose aspirin (LDA) and heparin and or steroids [49, 50]. A prospective study was carried out in two centers, patients in one of which were treated with prednisone and LDA (control group, *n* = 29) and the other used IVIg (experimental group, *n* = 53) [50]. The results showed that the birth weight of newborns in the experimental group was significantly higher than that in the control group, and the incidence of pregnancy-induced hypertension and gestational diabetes in the control group was significantly higher than that in the experimental group [50]. IVIg has the function of neutralizing autoantibodies, as a result of which it can be considered used in RPL patients with APS who have previously failed in other treatments [27, 50, 51].

Nevertheless, there are some negative findings. In a randomized controlled study, Triolo et al. confirmed that low-molecular-weight (LMW) heparin and aspirin showed a higher live birth rate than IVIg in patients with RPL associated with APS (84% vs. 57%) [52]. In another prospective study, 85 RPL patients with APS aged 18-39 years from 2002 to 2006, and these patients were randomly divided into LMW heparin plus the LDA group and IVIg group [53]. The live birth rate of the former was significantly higher than that of the latter (72.5% vs. 39.5%, *P* = 0.003) [53]. In another study, the control group received LDA plus heparin and placebo, while the experimental group received LDA plus heparin and IVIg (1 g/kg/month) [54]. No miscarriage occurred in either group [54]. The incidence of preeclampsia (44% vs. 11%) and preterm delivery (100% *vs.* 33%) in the experimental group was slightly higher than that in the control group. The incidence of fetal distress (0 vs. 33%), fetal growth restriction (14% vs. 33%), and neonatal admission to the neonate intensive care unit (NICU) (14% vs. 44%) in the experimental group was mildly lower than that in the control group. However, there was no statistical difference about these outcomes [54].

These studies have changed a lot depending on the dose of IVIg, the time of administration, and the concomitant therapies used (e.g., LDA, LMW heparin, and prednisone). Although many studies believe that anticoagulant therapy is essential for APS patients, IVIg is effective for APS patients who do not receive heparin treatment. In other words, anticoagulation is not completely effective for APS patients. Some patients may benefit from IVIg and LDA or heparin because IVIg can neutralize APAs.

## 4. IVIg in Unexplained RPL

The reason IVIg can be used to treat unexplained RPL is that many patients with unexplained RPL have autoimmune disorders. The impact of IVIg on unexplained RPL has been reported by nine randomized placebo-controlled trials so far, but their findings are not consistent [27, 29, 30, 33–37, 55] (Table 2).

Previous randomized controlled trials (RCTs) indicated that the effect of IVIg on secondary RPL was considered slightly effective [26]. The difference between secondary RPL and primary RPL is the immune effect of male-specific minor histocompatibility (HY) antigens on the former [56]. The results of Cochrane systematic review with meta-analysis published in 2014 showed that IVIg had no better effect on RPL patients than placebo [57]. This meta-analysis, however, had only one outcome (live birth after 20 weeks of gestation) and only one subgroup (studies for cure) and did not include two recently published placebo-controlled trials [29, 30]. One year later, a systematic review and meta-analysis found that there was an RR of 0.92 for no live birth by IVIg for all participating patients [58]. The disadvantage of RCT studies in patients with primary RPL is that the number of samples is too limited to achieve meaningful differences after statistical analysis [59]. Wang et al. recently published a review [60]. The results showed that a RR for birth of primary RPL after IVIg treatment was 0.88 (95% CI 0.71–1.08; *P* = 0.20), and that of secondary RPL was 1.26 (95% CI 0.99–1.60; *P* = 0.06, one-sided test *P* = 0.03) [60]. IVIg has a moderately important therapeutic role in secondary immunologic RPL, according to their meta-analysis [60]. It was recommended that the application of IVIg in the secondary immunologic RPL can be considered [60].

As time passes, we have a deeper understanding of the etiology and pathogenesis of RPL. Recent studies have shown that many factors, such as genetic polymorphisms, pathogenic microorganisms, glycoproteins, and new immunopathogenic factors, may trigger RPL [61]. These pathogenic factors were not identified before 1994, so these factors in the series of unidentified RPL patients were not included in the study during that period. Some subgroups of unexplained RPL patients may benefit, but not all of them may benefit from IVIg. Therefore, the lack of awareness of unexplained RPL pathogenesis hinders people from conducting a well-controlled clinical study.

## 5. IVIg in RPL Patients with Cellular Immunity Abnormalities

### 5.1. IVIg in RPL Patients with Th1/Th2 Ratio Abnormalities

Yamada et al. confirmed that the abundant IVIg administration induced the expression of cytokines [Interferon *γ* (IFN-*γ*), tumor necrosis factor-alpha (TNF-*α*), interleukin-4 (IL-4), IL-10] in peripheral blood and reduced Th1/Th2 lymphocyte ratios [62]. Szereday et al. concluded that when lymphocytes from RPL patients and polyclonal IgG were cocultured, the expression of cytokines changed [63]. In essence, the expression of IL-12 (Th1-type reaction) decreased, while that of IL-10 (Th2-type reaction) increased [63]. Because of this result, an in vitro study of RPL patients showed that the binding capability of activated T cells and extracellular matrix (including collagen IV, fibronectin, and elastin) increased, and this phenomenon can be neutralized by IgG [64]. These results indicate that the placenta's extracellular matrix can absorb and affect the immune response in a way that improves the outcome of pregnancy [64].

Thirty-two patients were treated with IVIg (experimental group) in a sample of forty-four RPL women, and the other twelve patients were known as the control group [65]. The Th1/Th2 ratio in the experimental group decreased significantly at the end of treatment (*P* < 0.0001), twenty-eight cases (87.5%) in the experimental group had live birth, and only five cases (41.6%) in the control group delivered live fetuses successfully [65]. The IVIg administration decreased Th1 cells and led to a shift toward Th2 cells in RPL patients with APS [66]. These results showed that treatment with IVIg tends to be an important therapeutic method that can improve the pregnancy success rate through a change to Th2 reactions.

### 5.2. IVIg in RPL Patients with Treg/Th17 Ratio Abnormalities

There is strong evidence that IVIg enhances the suppressive activity of Treg cells [67, 68]. The research suggested that the mixture of IVIg in the Treg cell culture system increased the expression of forkhead box protein P3 (FOXP3), IL-10, and transforming growth factor (TGF)-*β* and induced the suppressive effect of Treg cells [68]. The combining ability of Treg cells to IVIg is stronger than that of conventional T cells [67], which shows that Treg cells can be easily changed by IVIg. Furthermore, IVIg can increase the number of existing Treg cells, rather than induce Treg cells from naive T cells [69]. The number of Treg cells increased considerably in the early stage of pregnancy [70, 71]. As a result, the promotion of IVIg on Treg cells benefited RPL patients whose Treg cells decreased.

The number and differentiation of Th17 cells decreased after adding IVIg to a human CD4^+^ T cell culture system [72]. Since IVIg does not contain anti-IL-17 antibodies, its effect has nothing to do with IL-17 neutralization. IVIg decreased Th17 cell proliferation and IL-17 production, and this function was not regulated through anti-IL-17 antibodies [72, 73]. When IVIg was added to CD4^+^ T cells without antigen-presenting cells (APCs), the function of Th17 cells immediately decreased, including the production of inflammatory cytokines [74]. Sha et al. [75] indicated that IVIg combined with hCG may inhibit the differentiation of Th17 cells in pregnant females with unexplained RPL and may increase the differentiation of Treg cells. They believed that Treg cells improved the mother's immune tolerance so that the embryo could develop to full term [75]. IVIg diminished the differentiation of naive CD4 T cells into Th1 and Th17 cells and concurrently promoted an increment of Treg cells [76]. In addition, Kim et al. [77] found that IVIg could play a role in women with activated immune effectors or inhibitory immune modifiers in vivo.

Another study included 94 patients with RPL whose blood was collected when the pregnancy test was positive [78]. On the same day, 44 patients received IVIg 400 mg/kg/month intravenously until 32 weeks after gestation. Another 50 RPL patients without IVIg were described as the control group. Once these patients reached 32 weeks, their peripheral blood was obtained again. The results showed that the number and function of Th17 cells decreased significantly in the experimental group, while Treg cells increased significantly. The numbers of successful pregnancies in the experimental group and the control group were 38 out of 44 (86.3%) and 21 out of 50 (42%), respectively. This experiment showed that IVIg could change the Treg/Th17 ratio in peripheral blood, improve Treg cell response, and reduce Th17 reaction in RPL patients with cellular immunity abnormalities. Based on the latest findings of the same team, the addition of IVIg stimulates Treg cells and decreases the reactions of exhausted Treg cells during pregnancy in RPL patients with cellular immune anomalies [79].

A number of empirical studies have presented the plausible actions of IVIg on Treg/Th17 ratio in other autoimmune diseases. Regarding Treg cells, several mechanisms were reported by many labs. For instance, one study involved 11 autoimmune rheumatic patients, and according to the results, the anti-inflammatory roles of IVIg treatment could result in increased Treg cells, which further promoted immune tolerance [80]. In patients with Kawasaki disease (KD), the protein and mRNA levels of FOXP3 undoubtedly contributed to the rapid recovery of patients following IVIg infusion [81]. The improvement of Treg cells discovered in the treatment of IVIg and regular medication could alleviate symptoms of patients with eosinophilic granulomatosis with polyangiitis (EGPA) [82]. Besides, IVIg treatment is regulatable via the immunomodulatory effect of dendritic cells (DCs), constructing an approach that increases antigen-specific and inhibitory Treg cells [83]. It was also shown that IVIg advanced Tregs through the generation of cyclooxygenase (COX)-2-dependent prostaglandin E2 (PGE2) in human DCs [84]. In studies involving animals, the number of platelets in mice increased following the administration of IVIg to immune thrombocytopenia (ITP) mice. In combination with lower levels of antibodies, Treg cells was produced by thyroid, and spleen was normalized [85]. IVIg can surge the ratio of Treg/Th17 through the IL-10 dependent pathway, which proved to be effective when treating collagen-induced arthritis (CIA) patients [86].

### 5.3. IVIg and NK Cells in RPL Patients

At the maternal-fetal interface, higher numbers and/or cytotoxicity of NK cells in peripheral blood can change to an inappropriate environment. Many studies have shown that CD56^+^ NK cells decrease in RPL women treated with IVIg [87–91]. In the research by Kwak et al. [87], the treatment of IVIg decreased the count and activity of CD56^+^ and CD56^+^/16^+^ NK populations and increased the rate of successful pregnancy in RPL women. In another research, NK cells were increased in pregnant (*n* = 187) and nonpregnant RPL women (*n* = 394), and NK cells significantly decreased after the first IVIg treatment [88]. The success rate for RPL pregnant women treated with IVIg was 92.3% [88]. Heilmann et al. [92] performed an assay that suggested a relationship between the decrease of NK cells and pregnancy outcome. The findings of this assay suggested that the count of NK cells (CD3^−^, CD56^+^, and CD16^+^) decreased in patients who delivered after IVIg treatment [92]. In RPL patients who received IVIg in the second trimester, the percentage of NK cells was significantly reduced [93]. Another study included 90 RPL patients with the IVIg administration [94]. 82.0% of these patients had a successful pregnancy outcome [94]. They indicated that with appropriate treatment, low-dose IVIg treatment was safe and effective for women with immunological miscarriage and characterized by increased NK cells [94]. In a study of women with RPL and increased expression of NK cells, live birth rates with IVIg were found to be 96.3 percent vs. 30.8 percent in women without IVIg administration [95]. In vivo, researchers found that IVIg restrained the increase in the count of CD44^bright^ subsets in uterine samples of unexplained RPL mice [96].

Expression percentiles of inhibitory CD94 on NK cells were significantly added after the administration of IVIg (*P* = 0.01) [97]. Mechanisms for the potential efficacy of IVIg treatment for RPL may include increased levels of CD94 and the following inhibition of cytotoxicity of NK cells. Clark et al. found that in the case of miscarriage, a soluble CD200 molecule, including IVIg, inhibits the cytotoxic response of NK cells [98, 99]. The CD56^bright^ subset of NK cells expressed at the fetal-maternal interface present receptors for CD200 that combine with CD200R2/3 on APCs, which subsequently stimulate CD4^+^ CD25^+^ Treg cells [100]. And these Treg cells can prevent miscarriage [101]. A large amount of IVIg was effective in fourteen unexplained RPL patients, and NK cells decreased from 40.9 ± 17.0% before injection to 15.0 ± 7.9% after injection [102]. IVIg was able to minimize NK cells in peripheral blood from 22 percent to 12 percent seven days after infusion in 14 patients with six or more abortions (*P* < 0.001) [103]. The decrease of NK cells in the successful group (−58.8%) seemed to be more significant than that in the failed group (−14.8%, *P* = 0.057) [103]. A study was conducted in 2019 including 78 patients with RPL whose peripheral blood was collected when the pregnancy test was positive [104]. After IVIg treatment, NK cell count and cytotoxicity decreased significantly after 32 weeks of gestation, and the levels of inhibitory receptors increased significantly, while those of activated receptors decreased significantly [104]. The pregnancy outcome of the experimental group was better compared to the control group (86.8% vs. 45%; *P* = 0.0006) [104].

NKT cells are a subgroup of thymus-dependent (T) cells that are essentially CD4^+^ but have NK lineage surface markers. NKT cells in peripheral blood (not in the decidua) are more likely to respond to Th2 during normal pregnancy [105]. In the same way as NK cells, NKT cells in the endometrium can induce inflammatory reactions, promote the production of Th1 cytokines, and lead to the development of RPL. IVIg is one of the therapeutic methods for patients with RPL associated with NK/NKT-like cell enlargement [106, 107]. A subgroup of women with high NKT, balanced by IVIg, showed a higher rate of successful pregnancy [108].

### 5.4. Cut-off Values for Cellular Immunity Abnormalities in RPL

To assess cellular immune abnormalities in patients with RPL, NK cell percentage and cytotoxicity assays as well as Th1/Th2 cell ratios in peripheral blood are needed. However, the published cut-off values of NK cell percentages in peripheral blood of RPL women differed from one study to another. Some experiments considered NK cell percent levels more than 12 percent of mononuclear cells to be a cut-off for the high NK cell expression related to bad pregnancy outcomes [109]. Another research considered the percentage to be more than 12.5 percent [110]. The percentage of NK cells, over 16.1 percent in Korean RPL women, is considered abnormal [111]. An Australian study sets the cut-off value to 18% [110]. The cut-off values of NK cell cytotoxicity and Th1/Th2 ratio were also various in different laboratories [24, 112], so there may be some deviation in analyzing the results of these experiments.

## 6. IVIg and Basophils in RPL Patients

Basophils are scarce granulocytes. Regardless of their low numbers, only 0.5% of leukocytes, they have crucial biological effects [113, 114]. Basophils are related to numerous allergic diseases, such as asthma, nasitis, and atopic dermatitis [115, 116]. Neonates and children born from the cesarean section have higher levels of basophils, so they face a higher likelihood of developing allergic diseases and asthma, which suggests the intrauterine origin of these inflammatory diseases [117]. Admittedly, basophils do not have the characteristics of traditional APCs [118–122]; however, they can play an immune role through the secretion of cytokines, and those tend to the Th2 type immune response and promote B cell differentiation. Reportedly, IL-3, granulocyte–macrophage colony-stimulating factor (GM-CSF), thymic stromal lymphopoietin (TSLP), IL-33, different toll-like receptor ligands, and allergen-specific IgE supply activating pathways to basophils [123–128].

IVIg has the capacity to promote the activation of IL-3–primed human basophils and reinforced IL-4 in basophils by combing with basophil surface-bound IgE [129]. According to the findings, IVIg can improve the Th2 immune response by acting on basophils. The finding showed that 10 mg/mL of IVIg could activate basophils, which was demonstrated by elevated expressions of CD69 and IL-4 [130]. Basophils are also able to decrease Th1 and Th17 responses by secreting IL-4 [131] and increase the expression of the inhibitory Fc receptor Fc*γ*RIIB on macrophages to reduce the inflammation reaction [132]. Through these mechanisms, IVIg could have a vital role for inducing basophils in the treatment of RPL.

## 7. IVIg and Autophagy in RPL Patients

Autophagy refers to an over activated programmed cell death, which is unlike apoptosis [133]. Autophagy is encountered across a variety of fields of the reproductive system, from zygote formation to parturition [134]. Autophagy has the capacity to enhance the ovarian dysfunction induced by insulin [135], and it occurs in the procedure of implantation [136] and decidualization [137]. Impaired autophagy can induce the production of IL-1*β* to promote inflammatory reaction at the maternal fetal interface, which could be the underlining reason for RPL with APS [138]. Autophagy inhibition of trophoblast cells elevates NK cytotoxicity through insulin-like growth factor-2 (IGF-2) and impairs trophoblast invasion via paternally expressed gene 10 (PEG10), and both these factors lead to RPL [139].

It was shown that IVIg treatment induced autophagy in peripheral blood mononuclear cells (PBMCs) and downregulated certain inflammatory mediators in peripheral blood, such as IL-8, TNF-*α*, IL-1*β*, and IL-17A [140]. Extensive analysis indicated that IVIg-increased autophagy solely arose in monocytes, DCs, and M1 macrophages, rather than M2 macrophages [140]. With regards to the mechanism, autophagy promoted via IVIg relies on F(ab′)2; however, it does not rely on sialylation and requires the endocytosis effect of IgG from innate cells [140]. Despite the use of large doses of IVIg to induce autophagy in patients with autoimmune illnesses, research has shown that autophagy can be promoted remarkablely when the concentration of IgG reaches 10 mg in healthy people [140]. Since IVIg is an aggregation of IgG extracted from healthy people, it may imply that IgG is crucial for maintaining normal immune homeostasis. These findings partly elaborate the action of IVIg infusion in the treatment of females with RPL by inducing autophagy.

## 8. The Heterogeneity of IVIg Studies in RPL

Patient populations included in the placebo-controlled studies and the treatment protocols performed were quite heterogeneous, and it is doubtful how much information is obtained from the integration of all women in the meta-analysis of these studies. Another confounding factor is the dosage of IVIg, which makes it difficult to compare different studies. There is currently no study on the IVIg dosage so that the optimum dosage cannot be determined. Placebos used in the study also affect outcomes, and some placebos may interfere with pregnancy outcomes. Albumin has been reported to be able to regulate ovarian hormone, which may be beneficial to patients with RPL [141]. Moreover, chromosomal abnormality is a common cause of abortion. IVIg can only work in patients with normal chromosomes. Thus, the incidence of aneuploid embryos may influence the findings of IVIg, having an illusion of futility, while the administration may be helpful in those pregnancies where it may help. To ensure that the embryo chromosome is normal, we need to improve the embryo karyotyping method earlier.

In alloimmune diseases affecting pregnancy outcomes, such as fetal alloimmune thrombocytopenia (FNAIT) [142] or haemolytic disease of the fetus [143], the usual dose of IVIg is much higher (about 1 g/kg/body weight), and the frequency is much higher (usually once per week). Over the years, the dosage applied in most of the protocols has not been according to dosage-result trials. A majority of people adhere to the protocol of the first report that used IVIg in FNAIT [144]. The setting of 1.0 g/kg was identical to the treatments for idiopathic thrombocytopenic purpura [145–147] and in newborns with alloimmune thrombocytopenia [148, 149]. Two empirical studies have investigated the effect of 0.5 g/kg/week and 1 g/kg/week on FNAIT with standard risk (no occurrence of intraventricular hemorrhage in the previous child), and according to the results, the former was similar to the latter [150, 151]. Besides, the therapeutic effect of 2 g/kg/week was also discussed [152, 153]; however, the setting of the control groups in both studies was not strict. Reportedly, antibodies passing through the placenta do not increase with an increase in maternal IgG [150], which suggested that the Fc receptor of the placental transport may be saturated. Additionally, IVIg is expensive and in short supply, and there is a probable risk of disease transmission. These problems indicate that it is essential to consider using the minimum dosage to achieve the therapeutic effect. A large-scale worldwide study could investigate this problem afterwards.

## 9. The Timing of Using IVIg

The timing of treatment with IVIg, either in preconception or after implantation, may also influence the outcome. Sapir et al. [154] found that IVIg treatment before conception improved the chance of live delivery in primary RPL patients, whereas in secondary RPL patients, IVIg following embryo implantation showed a higher success rate. On the contrary, it was found that IVIg treatment obtained by RPL patients before conception was not as helpful as administered after pregnancy was established [26]. In the meta-analysis by Wang et al. [60], similar findings were reported containing more number of RCTs. The RR for live birth in women who had IVIg before pregnancy was 1.69 in contrast to those who got a placebo (95% CI 1.33–2.14; *P* < 0.0001) [60]. It is worth noting that many RCT studies reported by Hutton et al. [26] and Wang et al. [60] were repetitive. In murine researches, the CD200 check point inhibitor (expressed in IVIg) can induce DCs, which might induce the Treg reaction if given before conception and decrease pregnancy failure rate [155]. These contrary studies influencing interventional treatment need an investigation for making sure the ideal administration timing.

## 10. Pharmacokinetics of IVIg in Pregnancy

After IVIg infusion, serum IgG concentrations were reported to show multicompartmental first-order kinetics to decrease rapidly for 1–7 days, followed by a more gradual rate of decrease [156]. As for low-molecular-weight immunoglobulin parts, including Fab and Fv, are absorbed through the kidneys, the majority of intact IgG has disappeared due to concentration-dependent catabolism [157]. Pregnancy did not play an important role in the weight-adjusted dose of exogenously received IVIg [158]. Since the half-life of IVIg is 18 to 25 days, it is suitable to use IVIg every 3-4 weeks. Time of termination of the IVIg administration and the need for further laboratory tests should be decided by the obstetrician according to the condition of the patient.

## 11. Side Effects of IVIg

The deficiencies of IVIg treatment are the possible risk of viral contagion, including HIV, hepatitis, and prions. There are no reports of viral transmission caused by IVIg therapy to date. Most patients have good tolerance to IVIg, with no more than 5% of patients having common side effects, such as headaches, myalgia, fever, chills, dizziness, nausea, and vomiting. In addition, treatment with IVIg does not cause premature birth [17]. Ruiz et al. [91] reported that the function of this therapy is to reduce the tumor-killing functions of NK cells. Therefore, the therapy should only be performed with the knowledge that it may induce some kind of cancer. The anaphylactic reaction was found in IgA deficient (<7 mg/dL) patients immediately after IVIg. The sugar stabilizer of IVIg is associated with renal dysfunction after high-dose IVIg infusion [159–161]. As a result, every patient receiving IVIg needs to be tested for IgA and renal function in advance. The use of IVIg did not lead to serious side effects on neonates [162]. The prenatal IVIg administration was not related to premature maturation or other abnormal neonatal immune response [163]. The extent of the side effects depends on the patient's age, the dosage of IVIg, and the intravenous infusion speed [164]. For patients with congenital heart disease or possible heart disease, IVIg should be injected gradually to prevent excessive liquid load and heart damage [165, 166]. For some patients with hereditary thrombotic tendencies, APS, or other thrombotic conditions, IVIg is a substance that promotes blood hypercoagulability and may cause thrombosis. There are reports of thrombosis after IVIg treatment [167]. Thus, for patients with hypercoagulable conditions, LMW heparin should be added during the application of IVIg.

## 12. Cost of IVIg

IVIg is expensive and can cost $800 per infusion at frequently used doses (400 mg/kg every 3-4 weeks) without additional hospitalization cost in China. However, the cost of this therapy can be balanced by a reduction in unsatisfactory maternal and fetal outcomes that often require expensive hospitalizations. As long as the logistical and financial issues involved in carrying out such a study are not resolved, IVIg may continue to be applied empirically, and the argument over its administration will go on.

## 13. Conclusions

In general, there is very limited evidence for the effect of IVIg in RPL, according to the literature. Two recent meta-analyses of IVIg use in RPL found no evidence of improved live birth rates [58, 168]. High-quality clinical studies are difficult to perform because neither couples nor obstetricians are willing to be randomly on placebo. According to the studies in the literature, a multicentered RCT study is needed to conclude definitely whether IVIg will help prevent RPL or not. Insulation of RPL with immune abnormalities could clearly increase the therapeutic effect of the IVIg administration in patients with RPL. It is recommended that IVIg infusion can be considered used before conception in RPL patients who have cellular immune abnormalities such as increased NK cell counts, NK cell cytotoxicity, or increased Th1/Th2 ratio, depending on the cut-off values of each hospital. There is a strong basis for providing this treatment to well-deserved patients who are eligible for treatment and who can afford the infusion or where the cost of treatment can be covered by medical insurance.

## Figures and Tables

**Table 1:** Possible mechanisms of intravenous immunoglobulin (IVIg) in the prevention of recurrent pregnancy loss (RPL).

	Mechanism
IVIg	Reduce autoantibodies
	Neutralize autoantibodies
	Decrease NK cell activity
	Control the secretion of cytokines
	Suppress the binding and activation of complement
	Modulate and blockade of Fc receptor
	Inhibit Fc*γ*RIIb signaling in macrophages
	Increase the clearance of pathogenic antibody by FcRn
	Suppress superantigens
	Regulate adhesion molecules on B- and T lymphocytes
	Increase apoptosis of activated cytotoxic lymphocytes
	Anti-idiotypic modulation
	Apoptotic mechanisms
	Formation of immune complexes

**Table 2:** Randomized controlled trials (RCTs) of intravenous immunoglobulin (IVIg) in women with unexplained recurrent pregnancy loss (RPL).

Authors (years)	The number of previous miscarriages	Primary/secondary RPL	The number of experimental group/control group	Placebo	Timing of IVIg treatment	Success rates (experimental group *vs.* control group)	Conclusions
NA (1994) [33]	Three or more	Primary	27/30	5% albumin	5-8 weeks	74% vs. 70% (*P* > 0.05)	The effect of IVIg in primary RPL could not be confirmed.
Christiansen (1995) [34]	Three or more	Secondary	34 total	1.5% albumin	After pregnancy was confirmed	52.9% vs. 29.4% (*P* > 0.05)	The effect of IVIg in secondary RPL was uncertain.
Coulam (1995) [37]	Two or more	Primary (*n* = 56) and secondary (*n* = 49)	47*/*48	0.5% albumin	500 mg/kg/month before conception	62% vs. 38% (*P* < 0.05)	IVIg was effective for RPL patients.
Perino (1997) [36]	Three or more	NA	22/24	5% albumin	25 g/day from 5-7 weeks	68% vs. 79% (*P* > 0.05)	IVIg cannot facilitate the progression of pregnancy.
Stephenson (1998) [35]	Two or more	Primary and secondary	32/30	Saline	500 mg/kg before conception	70% vs. 55% for secondary RPL	IVIg seemed to be effective for secondary RPL patients.
Jablonowska (1999)	Three or more	Primary and secondary	22/19	Saline	20 g every 3 weeks after	77% vs. 79% (*P* > 0.05)	There is no statistically
[55].					Heart beat was seen by ultrasound		Significant difference between IVIg and placebo.
Christiansen (2002) [27]	Four or more	Primary and secondary	29/29	1.5% albumin	800-1000 mg/kg when pregnancy test was positive	58% vs. 24% (*P* < 0.05)	IVIg may improve pregnancy outcome for secondary RPL.
Stephenson (2010) [29]	Three or more	Secondary	29/39	Saline	500 mg/kg preconception	70% vs. 63% (*P* > 0.05)	No treatment benefit was found.
Christiansen (2015) [30]	Four or more	Secondary	42/40	5% albumin	400 mg/kg when pregnancy test was positive	54.8% vs. 50% (*P* > 0.05)	IVIg did not enhance the live birth rate in secondary RPL patients.

NA: nonanalyzed.
